# Factors associated with delay in treatment-seeking behaviour for fever cases among caregivers of under-five children in India: Evidence from the National Family Health Survey-4, 2015–16

**DOI:** 10.1371/journal.pone.0269844

**Published:** 2022-06-16

**Authors:** Dhiman Debsarma, Jayanti Saha, Sagar Ghosh

**Affiliations:** 1 Centre for the Study of Regional Development, School of Social Sciences, Jawaharlal Nehru University, New Delhi, India; 2 Centre of Social Medicine and Community Health, School of Social Sciences, Jawaharlal Nehru University, New Delhi, India; 3 Government Shyamlal Pandaviya P.G. College, Morar, Gwalior, India; Freelance Consultant, Myanmar, MYANMAR

## Abstract

**Background:**

Fever is one of the common clinical symptoms found among children suffering from various illnesses. India carries a substantial burden of febrile illness among under-five children which heighten the risk of malnutrition, mortality and morbidity. This study aims to determine the factors associated with delay in treatment-seeking for fever among under-five children in India.

**Methods:**

A cross-sectional study was carried out using the large-scale nationally representative data from the National Family Health Survey (NFHS-4), conducted in 2015–2016. The data were collected by using four survey questionnaires i.e., Household Questionnaire, Woman’s Questionnaire, Man’s Questionnaire, and Biomarker Questionnaire. Delay in treatment-seeking was defined as taking a child for treatment after 24 hours of fever onset. Bivariate and multivariate logistic regression models were performed to assess the factors associated with delay in treatment-seeking behaviour for fever in under-five children.

**Results:**

In India, 31.12% (n = 7229) of the caregivers sought treatment for children after 24 hours of the onset of fever. Findings show no significant differences in delay in treatment-seeking behaviour by age groups and sex of children. Multivariate analysis revealed that the odds of delay in treatment-seeking behaviour of fever were higher among children from the poorest wealth quintile (AOR: 2.06; 95% CI: 1.85, 2.31), belonging to the scheduled tribe (AOR: 1.35; 95% CI: 1.24, 1.48), children who resided in rural areas (AOR: 1.14; 95% CI: 1.07, 1.22), children from the northeast region (AOR: 1.29; 95% CI: 1.14, 1.46), and children of caregivers who perceived distance to health facilities as a ‘big problem’ (AOR: 1.16; 95% CI: 1.09, 1.23).

**Conclusion:**

The study shows a high prevalence of delay in seeking treatment for fever among caregivers of under-five children in India. Delay in seeking treatment is associated with socio-demographic and socio-economic factors. Therefore, there is a need for intensified health promotion programs to sensitize caregivers on the importance of early health-seeking behaviour.

## Introduction

The United Nations International Children’s Fund (UNICEF) have pointed out that India records the highest global share of under-five deaths, with a total of 1.08 million deaths [1,2] and about two-thirds of all child deaths are related to preventable diseases such as diarrhea, malaria, pneumonia [3,4]. It is noteworthy to point out that in all these diseases fever is one of the common clinical symptoms found among children [5,6]. Childhood fever has been defined as the temperature at or above 100° F or 38°C a significant concern requiring medical advice and hospitalization [5,7]. Even different studies have used fever as the proxy measure on behalf of diseases like malaria, and dengue because its diagnostic test is costly and difficult while conducting a large-scale survey especially in the rural and remote areas [8–10]. According to the National Family and Health Survey (2015–16), in India, the prevalence of fever among under-five children was 12.9% [11]. But surprisingly, only 73.2% of the total children received medical treatment for fever. This reflects that India carries a substantial burden of febrile illness among under-five children which may heighten the risk of malnutrition, mortality and morbidity [11].

The children’s treatment-seeking behaviour completely depends upon their parents who are the foremost and primary caregivers of the children [12]. Since the children are unable to navigate and express the severity of the illness, their health vulnerabilities depend upon their caregivers’ perception of and treatment-seeking behaviour. Therefore, timely and appropriate treatment-seeking behaviour of caregivers is essential to reduce children’s health vulnerabilities [13]. Studies highlighted that early treatment-seeking for childhood fever morbidity would increase the chances of disease detection at the initial stage which may eventually help to stop the progression and severity of the illness [14].

A plethora of studies documented that the treatment-seeking behavior of the child is affected by numerous socio-demographic and economic factors such as the age of the child, sex of the child, maternal age, maternal education, wealth quintile, cultural beliefs on the illness, availability of health facilities, and occupation of the caregiver, place of residence, and proximity to health care facilities [4,8,15–23]. Various studies also dealt with the associated factors of utilization of health care, knowledge and fever case management practices of the child’s caregiver [5,24–27].

In India, few studies were conducted regarding the determinants of the treatment-seeking behaviors for fever among the caregivers of the under-five child [28–30]. An investigation carried down in Gujarat shows a significant association between the treatment-seeking behavior of the caregiver of the child and socio-economic status of the mother, joint family structure, exposure to mass media, level of education, and gender differences of the child [28].

Delay in the treatment-seeking behaviour for fever among caregivers of under-five children is a matter of serious concern causing child morbidity and mortality [8,31]. However, in India there are no robust evidences in understanding the determinants of treatment-seeking behaviour of the caregivers of the under-five children suffering from fever. Therefore, this study attempts to identify the factors associated with delay in treatment-seeking among caregivers for their children under-five years. The findings of this study would provide vital inputs that will potentially necessitate designing proper interventions to reduce delay in treatment-seeking behavior of the caregivers of under-five children which may further reduce the potential rate of preventable morbidity and mortality of children.

## Materials and methods

### Data source

A cross-sectional study was carried out using the large-scale, multi-round nationally representative data from the fourth round of the National Family Health Survey (NFHS-4), conducted in 2015–2016. A detailed account of the sampling procedure of the survey is demonstrated in the national report of the International Institute for Population Sciences (IIPS), 2017 [11]. The survey used four types of questionnaire which includes: Household, Biomarker, Man, and Woman. The household questionnaire covered the socio-economic information of the participant. The Biomarker questionnaire covers Clinical, Anthropometric, and Biochemical (CAB) components, anemia, hypertension, HIV, and blood glucose data for women aged 15–49 and men aged 15–54. The men questionnaire includes man’s background characteristics, media exposure, marriage, employment, number of children, presence at antenatal care visits, contraceptive knowledge and use, fertility preferences, nutrition, sexual behaviour, attitudes toward gender roles, HIV/AIDS, tobacco and alcohol use. The women’s questionnaire also covered background characteristics of the women, reproduction, family planning, maternal and child health practices which include: antenatal care; delivery care; postnatal care, treatment of fever and diarrhea. The NFHS-4 survey covered 699,686 women aged 15–49 years in 601,509 households across all the states and union territories (UTs) of the country (IIPS, 2017). The data collector gathered the child’s health-related information details from the mother using the well-prepared biomarker questionnaire. The interviewer recorded the mother/caregiver’s responses whether their children had a fever within the last two weeks prior to the survey; time duration and place of first sought advice or treatment.

### Study participants and sample size

**Fig 1:**
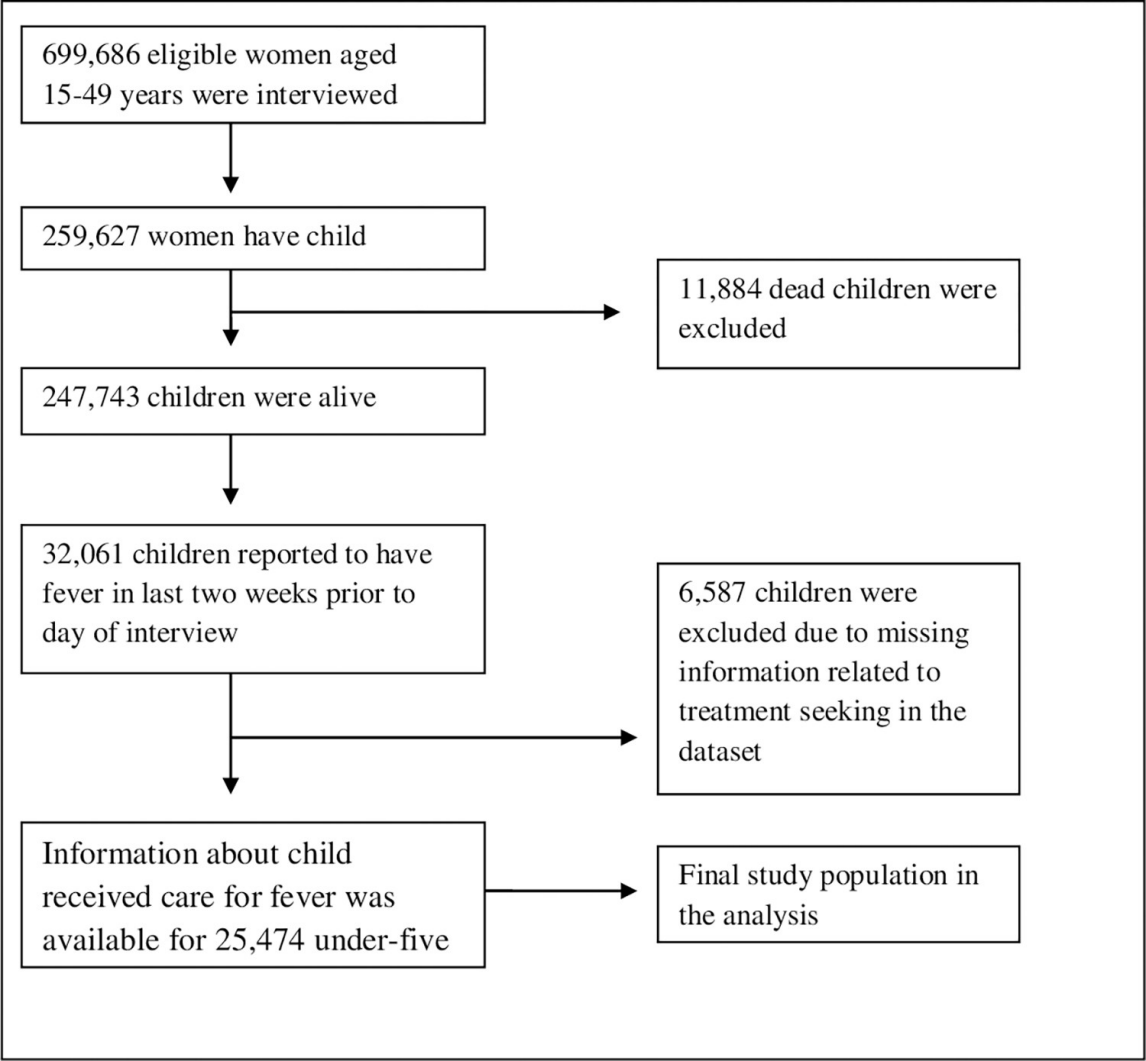
Selection of study participants, NFHS-4.

The NFHS-4 provided information for 259,627 children born to women aged 15–49 years in the past five years preceding the survey. Out of these, 247,743 children were alive and 11, 884 children were dead. In the last two weeks prior to the survey 32, 061 children reported experiencing fever. However, in this study 6587 children were excluded due to missing information regarding treatment-seeking behaviour for fever in the dataset. Therefore, the final study sample for the analysis was 25,474 under-five children (Fig 1).

### Outcome variable

The outcome variable is “delay in treatment-seeking” defined as seeking treatment after 24 hours from the onset of fever of the children under-five by the caregiver [32].

### Explanatory variables

Based on the existing literature we included various socio-demographic factors as explanatory variables: age of child (0–11, 12–24, 25–36, 27–48, and 49–60 months), sex of child (male and female), place of residence (urban and rural), region (north, central, east, northeast, west and south), caste (scheduled caste, scheduled tribe, backward classes[OBC], other), religion(Hindu, Muslim, Christian and other), household wealth quintile (poorest, poorer, middle, richer and richest), age of the mother (15–24, 25–34, and 34–49 years), level of mother education (no education, primary, secondary and higher secondary), place of treatment (public, private, community centre), distance to health facility (no problem, not a big problem, big problem).

### Statistical analysis

In this study, descriptive statistics were carried out to understand the distribution of socio-economic and demographic characteristics of the study participants. Pearson chi-square test was performed to examine the relationship between delays in treatment for child fever and selected categorical socio-economic and demographic variables. A multivariate logistic regression analysis was performed to assess the factors associated with delay in treatment-seeking behaviour for fever in under-five children. In the multivariate logistic regression analysis model building, the backward stepwise elimination was used. All the statistical analyses were performed using version 14 (StataCorp LP, College Station, Texas, USA).

### Ethical statement

The data used in this study was obtained from the National Family Health Survey (NFHS-4). The large datasets are accessible from the Demographic Health Survey (DHS) program at https://dhsprogram.com/data/available-datasets.cfm. The Ethics Review Board granted the ethical approval of the NFHS-4 at the International Institute for Population Sciences (IIPS), Mumbai, India, before conducting the survey. Written informed consent was sought from participants before starting the interview. The NFHS-4 is an anonymous publicly available dataset with no identifiable information about the survey participants. Therefore, no separate ethical statement is required for this work.

## Results

### Background characteristics of the study participants

Among 25474 living under-five children, 32. 28% of children belonged to the age group of 0–12 months and 54.07% were males. The majority of the children (73.70%) resided in the rural areas, affiliated with the Hindu religion (70.37%), belonged to the Other Backward Classes (47.19%), belonged to poor families (25% in the poorest wealth quintile and 22.77% in the poorer wealth quintile), resided in the central region (35.68%) of India. A substantial proportion of mothers (55.92%) was 25–35 years, and 28.36% of the mother had no formal education.

### Treatment-seeking behaviour of caregivers of under-five children with fever by background characteristics in India

Table 1 illustrates the treatment-seeking behaviour of caregivers of under-five children with fever in India. Out of total respondents, 71.62% of the caregivers sought health care for children before 24 hours of noticing the onset of fever whereas 31.12% of children waited up to 24 hours (i.e., delayed care) to get treatment. The study found that the prevalence of delay treatment-seeking among caregivers for children to be comparable across all age groups. Delay in treatment-seeking for child fever episode was higher among the children aged 49–60 months (31.94%). The proportion of male and female children who received care after 24 hours later from the onset of fever was 30.92% and 31.37% respectively. A significantly higher proportion of caregivers from rural areas (33.21%) compared to those from urban areas (24.78%) sought treatment after 24 hours of the onset of fever, P<0.001. The findings of the study outstandingly revealed that with decreases in the gradient of the wealth quintile of household and the level of mother’s education, the rate of delay in treatment-seeking for child fever episode tend to decline substantially. Similarly, the caregiver who belonged to the Scheduled tribe (40.71%; P<0.001) and affiliated to Christian religion (39.51%; P<0.001) approached the health facility to seek care for their child with fever episodes after 24 hours of the onset of fever. Furthermore, the percentage of delay treatment-seeking widely varied across the regions of the country. The north-east region comprises the highest (41.96%) in delay in child treatment-seeking, followed by eastern (35.11%), central (27.81%) and southern (25.24%) states. Thirty seven percent (37.00%), 28.62% and 29.03% of the caregivers sought advice or treatment after 24 hours of the onset of fever from public health facilities, community health workers and private health facilities, respectively.

**Table 1:** Demographic, socio-economic and regional differential in treatment-seeking behaviour of caregivers of under-five children with fever in India, NFHS-4 (2015–16).

Variables	Timing of treatment				P-value
	Early Care (<24 hours)		Delay Care (> 24 hours)		
	Frequency	Percentage	Frequency	Percentage	
**India**	18,245	68.88	7,229	31.12	
**Age of child in months**					0.800
49–60	1,396	68.06	655	31.94	
27–48	2,143	69.31	949	30.69	
25–36	3,159	68.55	1,449	31.45	
12–24	5,155	68.74	2,344	31.26	
0–11	5,693	69.22	2,531	30.78	
**Sex of child**					0.446
Male	9,642	69.08	4,316	30.92	
Female	7,904	68.63	3,612	31.37	
**Place of residence**					<0.001
Urban	4,748	75.22	1,564	24.78	
Rural	12,798	66.79	6,364	33.21	
**Maternal age in years**					<0.001
34–49	1,515	67.27	737	32.73	
25–34	10,002	70.14	4,258	29.86	
15–24	6,029	67.27	2,933	32.73	
**Wealth quintile**					<0.001
Richest	2,881	79.32	751	20.68	
Richer	3,373	73.58	1,211	26.42	
Middle	3,798	69.28	1,684	30.72	
Poorer	3,942	65.82	2,047	34.18	
Poorest	3,552	61.38	2,235	38.62	
**Maternal education**					<0.001
No education	4,770	66	2,443	33.87	
Primary	2,654	66.87	1,315	33.13	
Secondary	8,181	69.51	3,589	30.49	
Higher secondary	1,941	76.96	581	23.04	
**Caste**					<0.001
Scheduled Castes	3,609	70.13	1,537	29.87	
Scheduled Tribes	2,106	59.29	1,446	40.71	
Other Backward Classes	7,685	71.18	3,112	28.82	
Others	3,447	69.57	1,508	30.43	
**Religion**					<0.001
Hindu	12,347	68.36	5,716	31.64	
Muslim	3,560	70.93	1,459	29.07	
Christian	932	61.76	577	38.24	
Others	707	80.07	176	19.93	
**Place of first treatment**					<0.001
Public	4,296	63	2,523	37	
Private*	11,847	70.97	4,847	29.03	
Community centre	1403	71.38	558	28.62	
**Region**					<0.001
North	3,582	68.24	1,667	31.76	
Central	6,801	72.19	2,620	27.81	
East	3,197	64.89	1,730	35.11	
Northeast	1,318	58.04	953	41.96	
West	1,090	71.62	432	28.38	
South	1,558	74.76	526	25.24	
**Distance to health facility**					<0.001
No problem	5,970	73.86	2,113	26.14	
Not a big problem	5,542	67.95	2,614	32.05	
Big problem	6,034	65.34	3,201	34.66	

*P-values* denote the level of significance of Pearson’s chi-square statistic.

*Private health facilities comprise of private hospital, pharmacy/drugstore, clinic, paramedic, and AYUSH, NGOs or trust hospital/clinic.

### Factors associated with delay in treatment-seeking behavior for child fever

Table 2 illustrates the results of univariate and multivariate logistic regression models to examine the factors associated with delay in treatment-seeking behavior for under-five children among caregivers in India. The unadjusted analysis revealed that children living in rural areas were associated with higher odds of delay in treatment-seeking (Unadjusted OR: 1.51, 95% CI: 1.42–1.61) compared to those living in urban areas. Children of mothers in the age range of 25–34 years (Unadjusted OR: 0.88, 95% CI: 0.80–0.96) were less likely to delay the treatment than those children of mothers aged 34–49 years. Children in the poorest wealth quintile were 2.4 times more likely to be treated after 24 hours of the onset of fever as compared to those children in the richest wealth quintile. Delay in seeking treatment was found to be significantly higher among the children who belonged to the Scheduled tribe (Unadjusted OR: 1.61, 95% CI: 1.47–1.76) than those from Scheduled caste. Christian children were 34% more likely to seek treatment after 24 hours. Caregivers had a reduced chance of 32% (Unadjusted OR: 0.68, 95% CI: 0.61–0.76) of seeking delay treatment if they took a child to a community centre compared to a public health facilities. Similarly, caregivers of the children who resided in the North-East (Unadjusted OR: 1.55, 95% CI: 1.40–1.72) and East region (Unadjusted OR: 1.16, 95% CI: 1.07–1.26) were more likely to delay in seeking treatment than those living in the north region of the country.

**Table 2:** Factors associated with delay in treatment-seeking behaviour of caregivers of under-five children with fever in India NFHS-4, (2015–16).

Variables	Crude OR	95% CI		Adjusted OR	95% CI	
**Age of child in months**						
49–60	1.00			1.00		
37–48	0.94	0.84	1.06	0.90**	0.82	0.98
25–36	0.98	0.87	1.09	0.93	0.85	1.01
12–24	0.97	0.87	1.08	0.89**	0.82	0.96
0–11	0.95	0.85	1.05	0.82**	0.75	0.9
**Sex of child**						
Male	1.00			1.00		
Female	1.02	0.97	1.08	1.00	0.95	1.05
**Place of residence**						
Urban	1.00			1.00		
Rural	1.51**	1.42	1.61	1.14**	1.07	1.22
**Maternal age in years**						
35–49	1.00			1.00		
25–34	0.88**	0.80	0.96	0.93	0.85	1.02
15–24	1.00	0.91	1.10	1.06	0.96	1.16
**Wealth quintile**						
Richest	1.00			1.00		
Richer	1.38**	1.24	1.53	1.32**	1.20	1.45
Middle	1.70**	1.54	1.88	1.44**	1.31	1.59
Poorer	1.99**	1.81	2.19	1.67**	1.50	1.85
Poorest	2.41**	2.19	2.66	2.06**	1.85	2.31
**Maternal education**						
No education	1.00			1.00		
Primary	0.97	0.89	1.05	1.00	0.92	1.08
Secondary	0.86**	0.80	0.91	1.04	0.97	1.11
Higher	0.58**	0.53	0.65	0.97	0.87	1.08
**Caste**						
Scheduled Castes	1.00			1.00		
Scheduled Tribes	1.61**	1.47	1.76	1.35**	1.24	1.48
Other Backward Classes	0.95	0.88	1.02	1.03	0.96	1.10
Others	1.03	0.94	1.12	1.14**	1.05	1.24
**Religion**						
Hindu	1.00			1.00		
Muslim	0.89**	0.83	0.95	0.92*	0.85	0.98
Christian	1.34**	1.20	1.49	0.72**	0.63	0.83
Others	0.54**	0.45	0.64	0.47**	0.40	0.55
**Place of first treatment**						
Public	1.00			1.00		
Private	0.70**	0.66	0.74	0.79**	0.74	0.83
Community centre	0.68**	0.61	0.76	0.70**	0.63	0.77
**Region**						
North	1.00			1.00		
Central	0.83**	0.77	0.89	0.73**	0.68	0.78
East	1.16**	1.07	1.26	0.84**	0.77	0.91
Northeast	1.55**	1.40	1.72	1.29**	1.14	1.46
West	0.85*	0.75	0.97	0.79**	0.71	0.89
South	0.73**	0.65	0.81	0.73**	0.66	0.80
**Distance to health facility**						
No problem	1.00			1.00		
Not a big problem	1.33**	1.25	1.43	1.10**	1.03	1.17
Big problem	1.50**	1.40	1.60	1.16**	1.09	1.23

**p < 0.01.

*p < 0.05.

COR: Crude odds ratio, AOR: Adjusted odds ratio; CI: Confidence interval.

Private health facilities comprise of private hospital, pharmacy/drugstore, clinic, paramedic, and AYUSH, NGOs or trust hospital/clinic.

Children aged 0–11 months were less likely to be delayed in seeking care (Adjusted OR: 0.82, 95% CI: 0.75–0.90), compared to the children aged 49–60 months. Children residing in rural areas had a higher likelihood of delay in seeking treatment (Adjusted OR: 1.14, 95% CI: 1.07–1.22) than the urban counterparts. Children from the poorest wealth quintile were more likely to have delayed treatment (Adjusted OR: 2.06, 95% CI: 1.85–2.31) compared to the children from the richest wealth quintile. The likelihood of delay in treatment-seeking was 35% higher among the Scheduled tribe (Adjusted OR: 1.35, 95% CI: 1.24–1.48) as compared to the Scheduled Caste. Muslim children had 9% reduced odds of having a delay in treatment-seeking (Adjusted OR: 0.92, 95% CI: 0.85–0.98) as compared with the children belonging to the Hindu religion. Children from the northeast region were more likely (Adjusted OR: 1.29, 95% CI: 1.14–1.46) to delay the treatment than children from the country’s north region. But the children from the south (Adjusted OR: 0.73, 95% CI: 0.66–0.80) and central region (Adjusted OR: 0.73, 95% CI: 0.68–0.78) were less likely to delay the treatment as compared to children from the north region of the country. Delay of treatment-seeking behaviour of caregivers for fever decreased by 21% and 30% who sought treatment from private health facilities (Adjusted OR: 0.79, 95% CI: 0.74–0.83) and community centre (Adjusted OR: 0.70, 95% CI: 0.63–0.77) compared to those caregivers who took a child to a public health facility. The odds of delay in treatment-seeking were increased by 16% among children of those caregivers who reported distance to a health facility was a ‘big problem’ than those who perceived distance to a health facility was ‘not a problem’. The findings of our study reported that distance to the health facility is strongly associated with delay in seeking treatment where caregivers reported distance to a health facility was a ‘big problem’.

## Discussion

The study identified that delay in treatment-seeking for fever among the caregivers of the children under-five in India was prevalent (31.12%). Despite the intensive implementation of programs aimed at educating mothers of child-bearing age on issues surrounding child health, the prevalence of delay in treatment-seeking behaviour is quite high not only in rural areas but also in urban areas in India [33,34]. The factors significantly associated with delay in treatment-seeking among the caregivers were children age, place of residence, wealth quintile, caste, region, place of first treatment and distance to the health facility.

The findings in this study revealed that 31.12% of caregivers sought delayed treatment for the under-five children throughout the country. Previous studies carried out in Zambia (42%), North Central Nigeria (43%), Eastern Uganda (55.2%), Tanzania (55.4%) and Ethiopia (61.3%) also found high prevalence of delay in treatment-seeking for fever among the caregivers of the children under-five [8,17,35–37]. The children who resided in rural areas and belonged to the poor wealth quintile delay more in seeking treatment for fever. The findings in the present study are consistent with a study carried out in Tanzania [37]. Our study also reveals that the prevalence of seeking delay treatment is higher for those if the caregivers took a child to public health facilities. This is in line with a study conducted in Zambia [8].

This study has identified a number of concerning factors associated with delayed treatment-seeking behaviour. Multivariate analysis of this study has found that the age groups of the child were significantly associated with delay in caregivers’ treatment-seeking behaviour of child under-five. This might be due to the fact that the caregivers perceive the younger children especially the infant who need special and prompt care [18]. This finding is in line with other studies conducted in Nigeria [18], Tanzania [37], Ethiopia [38] Kenya [39] and Bangladesh [40]. But a study conducted in Zambia observed that treatment-seeking behaviour for fever was similar across all age groups [8].

Consistent with the findings of previous studies, our study also found that the wealth quintile has a strong association with the delay in treatment-seeking behaviour [4,28,41]. Children in the poorest wealth quintile were 2.4 times more likely to be treated after 24 hours of the onset of fever than the children in the richest wealth quintile. This is probably because poor households are unable to afford the costs of transportation and medications [35]. But this finding is inconsistent with the previous study conducted in Malawi [42]. It was noticed that the caste of the caregivers was significantly associated with delay in treatment-seeking for under-five children; children from Scheduled tribes were more likely to delay in seeking treatment. The results reported from this study suggest that the tribal population is generally found in the rural and remote locations where the health care services is still severely underdeveloped as a result affecting the tribal caregivers to take prompt action for their children before 24 hours of the onset of fever [43].

The place of residence of the caregiver is a significant factor contributing to delay in seeking treatment. Compared to the urban areas, the caregivers living in rural areas tend to reach health facilities after 24 hours of the onset of fever. This variation may be possible due to the distance and lack of appropriate health care facilities close to their residence in the rural areas [44,45]. This finding corroborated with studies conducted in other settings like Zambia [8] and Ethiopia. [46]. However, another study done in Ethiopia highlighted that the caregivers were more likely to seek treatment for under-five children in the rural areas than their urban counterparts due to the active participation of community health workers [17]. Moreover, the study reported caregivers’ perception of distance to health facility significantly contributed to delay in treatment-seeking. Our finding is consistent with other studies [21,36,37,47]. It might be due to the fact that the challenges of the caregivers to access health facilities increases when they report distance to health facility was a ‘big problem’. Long distance to health care facilities may also increase the out-of-pocket expenditure to pay transportation costs to and from the health facility which may further demotivate the caregivers to seek early treatment [37]. A higher proportion of respondents who used public health facilities sought treatment after 24 hours compared with other health facilities. It might be due to the fact that the private health facilities and community centres are densely located in proximity of the rural and urban areas, on the contrary the public health facilities are very limited. Hence, the people can easily access and sought treatment from private health facilities and community centres at any point of time. This finding is similar with the study reported in rural Tanzania [48]. Our study revealed that children from the North Eastern region were 29% more likely to delay the treatment as compared to children from the north. It is important to note that the complex physical topography of North East India is not conducive to transportation activities and takes a long time to travel, making it difficult to deliver and utilize health care services [49]. Similarly, our findings are parallel with another study conducted in Nigeria which also highlighted that geographical regions influence the treatment-seeking behaviour of the caregiver [18].

Our present study has some limitations. This study relied on self-reported recall answers hence the potential for recall bias as well as reporting bias cannot be ignored. The dataset does not contain information on specific types of fever and perceived severity of illness for which the study cannot deal with the treatment-seeking behaviour based on the different types of fever and perceived severity of illness. As the study deals with the secondary data it could not take into consideration the cultural beliefs and attitude towards the provider which also largely contributes to determine the treatment-seeking behaviour therefore, further qualitative research is required to improve upon these particular limitations. Despite these limitations, this study reveals various factors of delay in treatment-seeking of fever among children under-five which may be helpful for the policy makers to implement appropriate intervention to increase prompt treatment for fever.

## Conclusion

Our study revealed a high prevalence of delay in treatment-seeking for fever among caregivers of under-five children in India and it is associated with various socio-demographic and socio-economic factors such as the age of the child, place of residence, wealth quintile, caste, religion, region, place of treatment and caregivers’ perception on the distance to the health facility. As fever is the common symptom of illness, seeking early treatment would increase the chances of disease detection at the initial stage which may eventually help to stop the progression and severity of the illness. The policymakers should pay attention to formulate effective intervention to sensitize caregivers on the importance of early treatment-seeking behaviour. Community awareness program should be encouraged particularly in rural areas to make people aware of the necessity to take prompt action to seek care in the early stage.
